# Differences in COVID-19 Vaccination and Experiences among Patients with Hypertension in Colombia and Jamaica during the COVID-19 Pandemic

**DOI:** 10.3390/ijerph21101356

**Published:** 2024-10-15

**Authors:** Jacqueline P. Duncan, Siyi Geng, Carene Lindsay, Trevor S. Ferguson, Katherine T. Mills, Jose Patricio Lopez-Lopez, Hua He, Paola Lanza, Allison N. Marshall, Makeda J. Williams, Veronica Tonwe, Mabel Reyes, Alfonso Campo, Patricio Lopez-Jaramillo, Marshall K. Tulloch-Reid

**Affiliations:** 1 Department of Community Health and Psychiatry, The University of the West Indies, Kingston 7, Jamaica; 2 Department of Epidemiology, Tulane School of Public Health and Tropical Medicine, Tulane University, New Orleans, LA 70112, USA; sgeng1@tulane.edu (S.G.); kmills4@tulane.edu (K.T.M.); hhe2@tulane.edu (H.H.); planza@tulane.edu (P.L.); amarshall@tulane.edu (A.N.M.); 3 Epidemiology Research Unit, Caribbean Institute for Health Research, The University of the West Indies, Kingston 7, Jamaica; carene.lindsay@uwimona.edu.jm (C.L.); trevor.ferguson02@uwimona.edu.jm (T.S.F.); marshall.tullochreid@uwimona.edu.jm (M.K.T.-R.); 4 Masira Research Institute, Universidad de Santander (UDES), Bucaramanga 680003, Colombia; josepatriciolopez@gmail.com (J.P.L.-L.); ma.reyes@mail.udes.edu.co (M.R.); jplopez@gmail.com (P.L.-J.); 5 Center for Translation Research and Implementation Science, National Heart, Lung and Blood Institute (NHLBI), NIH, Bethesda, MD 20892, USA; willimak@mail.nih.gov (M.J.W.); veronica.tonwe@nih.gov (V.T.); 6 Department of Epidemiology, Faculty of Medicine and Health Science, Universidad de Santander (UDES), Valledupar 200001, Colombia; epid.campo@gmail.com

**Keywords:** COVID-19, vaccine hesitancy, health services, healthcare, Jamaica, Colombia

## Abstract

During the COVID-19 pandemic, hypertensive patients had increased infection and healthcare disruption in many low- and middle-income countries (LMICs) with limited vaccine access. The objective of this report is to describe COVID-19 experiences and vaccination uptake among hypertensive patients in Colombia and Jamaica. A cross-sectional study of patients with hypertension was conducted in primary care clinics in both countries between 2021 and 2022. Trained interviewers used a telephone-administered questionnaire to assess COVID-19 experiences (infection, vaccination, and healthcare access). A total of 576 patients (68.5% female, mean age: 67.5 years) participated. Health service disruption affecting access to care was low (<10%). Compared to Jamaica, more participants from Colombia reported testing positive for COVID-19, having family members or friends testing positive, losing family members or friends due to COVID-19, and being vaccinated. In logistic regression models, adjusted for age, sex, education, and rural/urban clinic status, fear of COVID-19 (OR 2.7, 95% CI: 1.2–6.1) and residence in Colombia (OR 5.9, 95% CI: 2.4–14.6) were associated with higher vaccination uptake. Disparities in access to COVID-19 testing and diagnosis may have influenced these country differences including fear of COVID-19 and vaccine uptake. Other factors need to be better understood to prepare for future pandemic responses.

## 1. Introduction

The coronavirus disease 2019 (COVID-19) was declared a public health emergency of international concern on 30 January 2020. Non-pharmaceutical interventions (NPIs), such as border closures, curfews, mandatory face masks, and hand sanitization, were implemented in countries to stave off waves of the pandemic [1]. In low- and middle-income countries (LMICs), health sectors experienced direct and indirect consequences of the COVID-19 response, which likely contributed to excess mortality [2]. This includes disruptions of medication supply chains and health services [3,4], as well as increases in body mass index due to changes in diet and physical activity [5]. These negative effects likely had more severe impacts on persons living with chronic diseases, such as hypertension [6,7,8].

Colombia and Jamaica are middle-income countries in Latin America and the Caribbean (LAC). The first case of COVID-19 was confirmed on 6 March 2020 in Colombia and 10 March 2020 in Jamaica [9,10]. By 31 December 2022, Colombia (total population 52 million) had 6.3 million cases and cumulative excess deaths of 365 per 100,000, while Jamaica (total population 2.9 million) had 158,000 cases and 192 per 100,000 cumulative excess deaths [11,12]. Both Colombia and Jamaica have a high prevalence of hypertension (24% and 34%, respectively) [13,14], which is a risk factor for severe COVID-19 and related mortality [15,16]. As a result, patients with hypertension were prioritized for vaccination and shielding. Despite the rapid development of COVID-19 vaccines, their distribution posed significant logistical challenges and barriers, including equitable distribution among countries and prioritization of high-risk populations.

By mid-2021, most high-income countries (HICs) had vaccinated a significant portion of their populations, while most LMICs had barely begun their vaccination campaigns [17]. Two years after the introduction of COVID-19 vaccines in LAC, approximately 26.6% of Jamaica’s population was fully vaccinated (defined as one dose of a one-dose vaccine or two doses of a two-dose vaccine) compared to 71.4% of Colombia’s population [11]. Vaccine uptake varies between and within other countries globally, including variations by ethnic groups [18,19], but the reasons for these differences are not fully understood. Further, the COVID-19 experiences of those in LMICs are not well documented despite the disproportionate burden of non-communicable diseases and the propensity for sustained disruptions in healthcare delivery and supply chain management. For patients with hypertension, COVID-19 healthcare disruptions could potentially include long appointment wait times and delayed access to care resulting in non-adherence to medication, uncontrolled hypertension, and cardiovascular complications.

During the needs assessment phase for the Caribbean and South America Team-based Strategy to Control Hypertension (CATCH) Study (ClinicalTrials.gov: NCT05405920), we assessed the COVID-19 experiences of hypertensive patients in Colombia and Jamaica and examined the factors associated with vaccine uptake. Conducting research in two countries in the LAC region with different healthcare systems and differences in vaccine uptake provided an opportunity to explore reasons for low vaccine uptake that may be relevant to other LMICs. The overall objective of this paper is to describe COVID-19 experiences, including infection, vaccination uptake, and access to care, for patients with hypertension in Colombia and Jamaica.

## 2. Materials and Methods

### 2.1. Study Design

The CATCH study is a cluster randomized trial to test the implementation and effectiveness outcomes of implementing and scaling up a team-based care strategy for blood pressure (BP) control in Colombia and Jamaica. As part of the needs assessment for CATCH, a cross-sectional study was conducted in four primary care clinics in Colombia and 10 primary care clinics in Jamaica between August 2021 and February 2022.

### 2.2. Recruitment

Convenience sampling was used to select clinics in both countries. Participant sampling differed between countries. In Jamaica, consecutive patients with hypertension were identified by reviewing medical records from the list of clinic attendees on non-communicable disease clinic days. In Colombia, patients with hypertension were randomly selected from electronic health records. Once eligible patients were identified, participants were invited to participate by phone by trained research staff. Sampling was stratified by urban/rural residence, sex, and age groups.

### 2.3. Data Collection

Interviewer-administered questionnaires were used to collect information on hypertension knowledge, lifestyle, medication adherence, and COVID-19 experiences. Questionnaires were administered by phone in both Spanish and English. Informed consent was obtained before the start of the interview. Copies of the consent form were provided to participants.The study protocol was approved by the Tulane University Institutional Review Board (New Orleans, LA, USA), the Institutional Bioethics Committee of Universidad de Santander (Bucaramanga, Colombia), and the University of the West Indies (UWI) Mona Campus Research Ethics Committee (Kingston, Jamaica).

### 2.4. Measurements

Validated questionnaires on hypertension knowledge, lifestyle, and medication adherence were used. Data were collected on COVID-19 experiences (COVID-19 infection, COVID-19 vaccination uptake, and health service disruption due to COVID-19). Data were also collected on demographic characteristics (age, sex, urban/rural location, education, and employment) and comorbidities. The questionnaire was piloted among 20 patients with hypertension (10 in Colombia and 10 in Jamaica) at two primary care clinics in Colombia and one primary care clinic in Jamaica.

### 2.5. COVID-19 Experiences

The outcome variable “COVID-19 vaccination” was measured by asking the question “Have you been vaccinated against COVID-19?” with dichotomized responses of “yes” or “no”. COVID-19 infection was determined by answering “yes” to either of the two options in the following question: “Since the COVID-19 pandemic began, have you experienced any of the following life events: (a) Testing positive for COVID-19? (b) Family member or close friend testing positive for COVID-19?” To assess fear of COVID-19 or complacency, we asked the respondents if “fear of contracting coronavirus if you leave the house” prevented them from seeing a doctor or obtaining their prescriptions.

### 2.6. Health Service Disruption

Health service disruption was measured using one item that asked participants if, during the COVID-19 pandemic, they had been unable to see a doctor or obtain prescription medications due to a list of reasons, from which participants could select multiple: clinics or hospital closed, pharmacy closed, less time to see a doctor or obtain medications due to curfew restrictions, fear of contracting COVID-19 if they leave the house, loss of insurance or government assistance, or trouble affording medications or healthcare costs.

### 2.7. Sociodemographic Variables

Self-reported sociodemographic characteristics were assessed, including age, gender, marital status, educational attainment (less than high school, high school, or more than high school), and employment status (employed/retired, unemployed, housewife, or disabled). Urbanicity (living in an urban or rural area) was assigned based on clinic location. Comorbid health conditions were assessed by self-report of diagnosis with the following conditions: diabetes, high cholesterol, kidney disease, stroke, heart attack/heart disease, and overweight/obesity. Mental health was assessed by self-report of diagnosis with depression and/or anxiety.

### 2.8. Sample Size

To determine the sample size required to construct a two-sided 95% confidence interval for the population proportion, we used PASS 15 (NCSS, LLC) software [20], employing the simple asymptotic formula with continuity correction to calculate the confidence interval [21,22]. This estimate was based on the assumption of stratification by sex and urban/rural status in each country. Our calculation indicated that a minimum of 72 participants per subgroup would be needed to detect a 20% response proportion with a 95% confidence interval ranging from 10% to 30%, requiring 288 participants per country.

### 2.9. Statistical Analysis

Analyses were performed using SAS 9.4 (SAS Institute, Cary, NC, USA). Mean and standard deviation for continuous variables and frequency and percentage for categorical variables are reported. Data were weighted to account for differences in the age, sex, and urban/rural area distribution of the study participants compared to national distributions of hypertension patients. Weights were determined from the joint distribution of age groups, sex, and residential areas for each country from clinic registers. In analyses that included participants from both countries, combined weights were applied.

Bivariate analyses were used to examine differences in COVID-19 experiences (testing positive, family member/friend positive, COVID-19 vaccination, and disruption of services impacting prescription attainment) and urban/rural status. Logistic regression was used to examine the factors associated with COVID-19 vaccination among hypertensive patients in both settings, as we explored reasons for differences in uptake. Unadjusted bivariate analyses and analyses adjusted for age, sex, education, rural/urban area, COVID-19 infection, fear of COVID-19 infection, and country are presented. A *p*-value < 0.05 was considered statistically significant.

## 3. Results

Two hundred and eighty-eight (288) participants with hypertension were surveyed in each country (n = 576, mean age: 67.5 ± 1.5 years). Characteristics of study participants by country are shown in Table 1. Most participants were female (68.5%), at least 60 years old (70.9%), employed or retired (69.7%), and from urban areas (68.1%). Colombian participants were older (mean age 66.5 vs. 62.5 years old, *p* = 0.001) and had a higher proportion of persons with more than high school education (30.3% vs. 17.2%, *p* = 0.011). Higher levels of unemployment (14.0% vs. 6.0%, *p* < 0.001) and “never married” status (34.0% vs. 5.5%, *p* < 0.001) were reported in Jamaica (Table 1).

### 3.1. Health Service Disruption

Table 2 shows the proportion of participants who experienced health service disruption based on urban/rural location. Few participants (<10%) were unable to see a doctor or obtain prescriptions due to clinic or pharmacy closure, curfews, having less time with a doctor, loss of health insurance, or healthcare costs. However, urban/rural differences in COVID-19 experiences were observed with greater negative impacts for participants in rural areas. For example, in rural areas, 13.0% and 10.4% of participants in Colombia and Jamaica, respectively, reported being “unable to see doctor or get prescription due to facility closure” compared to 1.6% and 2.1% in urban areas (Table 2).

### 3.2. COVID-19 Experiences

Overall, 15.8% of participants reported having tested positive for COVID-19 (24.2% in Colombia and 6.3% in Jamaica, *p* < 0.001 for difference) (Table 3). Participants in Colombia more commonly experienced having a family member or close friend test positive for COVID-19 (54.5% vs. 21.6%; *p* < 0.001), loss of a family member or friend due to COVID-19 (21.5% vs. 7.8%, *p* < 0.001), and having vaccination against COVID-19 (90.7% vs. 46.7%, *p* < 0.001) compared to participants in Jamaica. However, 44.4% of Jamaica’s participants who were not vaccinated were willing to be vaccinated if available (Table 3). Testing positive for COVID-19 was more common among urban participants in Colombia compared to rural participants (28.1% vs. 15.3%, *p* = 0.025), but no other significant urban/rural differences by country were observed.

Fear of contracting coronavirus was more common among Colombian participants (68.8% vs. 6.5%, *p* < 0.001) and among persons who were vaccinated or willing to be vaccinated against COVID-19 if available (47.0% vs. 4.7%, *p* < 0.001)).

### 3.3. Factors Associated with COVID-19 Vaccination Uptake

Table 4 shows the characteristics of the participants by vaccination uptake. Three hundred and ninety-seven participants were vaccinated (90.7% in Colombia and 46.7% in Jamaica; *p* for difference < 0.001). Vaccinated participants were older than unvaccinated participants (mean age 65.4 vs. 62.8 years old), more frequently married or in a common law relationship (56.8% vs. 35.2%), and had higher rates of overweight/obesity (47.4% vs. 32.3%). Other demographic characteristics and comorbidities were similar in both groups. Characteristics of COVID-19 vaccination status by country can be found in Tables S1 and S2 in the Supplementary Material.

The association between COVID-19 vaccination and sociodemographic characteristics, as well as COVID-19 experiences, is shown in Table 5. In unadjusted models, previous COVID-19 infection in a participant or family member, fear of COVID-19, and country of residence were associated with higher odds of vaccination. However, in a multivariable model adjusted for sociodemographic factors, only fear of COVID-19 (OR 2.71, 95% CI: 1.20–6.13) and country of residence (OR for Colombia vs. Jamaica 5.88 (95% CI: 2.38–14.56)) were independently association with vaccination against COVID-19.

## 4. Discussion

Our study found higher levels of COVID-19 infection, fear of COVID-19, and COVID-19 vaccine uptake in participants with hypertension in Colombia compared to Jamaica.

Since hypertension is a risk factor for severe COVID-19, these patients were prioritized for immunization across LAC [23]. While Mexico was the first country in Latin America to receive COVID-19 vaccines, Colombia and Jamaica were the first to receive COVID-19 vaccines through the COVID-19 Vaccine Global Access (COVAX) initiative in the Americas and the Caribbean, respectively [24,25]. A meta-analysis examining vaccine uptake in LAC reported high levels of vaccine uptake (pooled prevalence of 78%, 95% CI: 74.0%–82.0%) but only one Caribbean country/territory, Puerto Rico, was included in the pooled analysis [26]. Two studies from Colombia in this meta-analysis also showed high levels of vaccine intent (86% and 64%) [18].

In our study, COVID-19 vaccination was significantly higher in Colombia compared to Jamaica (90.6% vs. 46.7%, *p* for difference < 0.001). These findings are consistent with the COVID-19 vaccine uptake rates in the general population of both countries. By the end of the study period (1 February 2022), 61.2% and 22.0% of the general population in Colombia and Jamaica, respectively, were fully vaccinated [27,28]. As of 29 December 2022, Jamaica has the second lowest level of COVID-19 vaccination in the Caribbean with approximately 30% of the population receiving at least one dose of COVID-19 vaccine compared to 83% in Colombia [11]. Studies in other countries confirm suboptimal vaccination coverage among patients with hypertension (70 to 80%) and that comorbidities, such as hypertension, reduce the odds of vaccination [29,30,31].

Low uptake of the COVID-19 vaccine in LMICs has been attributed to global inequities in vaccine distribution and vaccine hesitancy [32]. Conceptual frameworks, such as the “three Cs model of vaccine hesitancy”, indicate that complacency or low perceived risk of vaccine-preventable diseases, as well as convenience (e.g., availability and acceptability) and confidence (or trust), are the main factors that influence vaccine hesitancy [33,34]. Surveys show that concerns about vaccine safety and the rapidity of vaccine development are the most common reasons for vaccine hesitancy in Jamaica [35,36]. In our study, 44.4% of Jamaica’s participants were willing to be vaccinated, if available, suggesting that a lack of availability and accessibility of COVID-19 vaccines may have contributed to the low uptake among hypertensive patients despite the establishment of fixed vaccination sites across the island. Structural factors, such as inequities in vaccine distribution and the digital divide (less internet access and use), also contributed to vaccine hesitancy among racial/ethnic minorities in developed countries [37]. Monitoring vaccine availability and uptake by socioeconomic status and other characteristics is important when addressing these inequities.

Meta-analyses show that female sex, younger age, unemployment, and lower educational attainment are associated with vaccine hesitancy [18,38]. Among persons with chronic diseases, healthcare provider recommendations and fear of side effects are common reasons for vaccine hesitancy [31,39,40]. Our analysis of patients with hypertension did not show any associations between vaccination and age, sex, education, or location (urban/rural). However, in multivariate models, vaccination was associated with fear of COVID-19 [adjusted OR 2.71 (95% CI: 1.20–6.13)], and fear of COVID-19 was significantly higher in Colombia compared to Jamaica (68.8 vs. 6.5%). Although COVID-19 infection is reported as a predictor for vaccine uptake in some studies, we did not find a statistically significant association between vaccination and history of COVID-19 infection when models adjusted for sociodemographic variables and country of residence [41,42]. Our findings suggest that while these factors should be considered when developing targeted interventions for vaccination of high-risk populations, issues related to vaccine availability, ease of access, and perceived risk of COVID-19 may be important considerations.

Fear of contracting COVID-19 may have been underestimated in our survey. We asked about fear leading to missed appointments or obtaining medications, specifically, those with fear of COVID-19 outside of those specific contexts will be missed. Structural differences in the healthcare systems in Jamaica and Colombia could influence patients’ concerns about missing appointments. However, our findings of the significantly lower levels of fear of contracting COVID-19 in Jamaica suggest a lower perceived threat of COVID-19 among Jamaica’s study population. Understanding and targeting the reasons for the lower perceived threat in LMICs such as Jamaica should be a priority for improving vaccine uptake. Inadequate access to diagnostic testing resulting in underdiagnoses of COVID-19 infections and deaths is one possible explanation for lower levels of fear of contracting COVID-19 in Jamaica and lower self-reported positive tests and loss due to COVID-19. Many LMICs had a shortage of rapid tests due to global inequities in rapid testing, and excess deaths during the pandemic are attributed to undiagnosed COVID-19 and inadequate diagnostic capacity [43].

COVID-19 antigen rapid testing was introduced in Colombia in July 2020 and Jamaica in September 2020 [44,45]. Colombia achieved one of the highest COVID-19 testing rates in Latin America with 33.9 million tests conducted by April 2022 [11]. COVID-19 testing was widely available, and by March 2022, the daily number of COVID-19 tests conducted in Colombia between January and March 2022 ranged from 2.9 to 13.6 tests per 1000 people, compared to 2.5 to 6.4 tests per 1000 people conducted in Jamaica during the same period [11]. In Jamaica, COVID-19 testing targeted hospitalized persons and symptomatic persons seeking care at public health facilities. Rapid testing was available in the private sector, but high costs made home testing unaffordable for most people. Approximately 320,000 tests per million people were conducted in Jamaica by March 2022. In Colombia, the number was more than double that of Jamaica, with 666,600 tests performed per million people [11,46]. This difference in access to testing in Jamaica and Colombia may also explain the finding that Colombia’s participants more commonly tested positive (24.2% vs. 6.3%, *p* < 0.001), had a family member or close friend test positive (54.5% vs. 21.6%; *p* < 0.001), and experienced losing a family member or friend to COVID-19 (21.5% vs. 7.8%, *p* < 0.001) compared to Jamaica.

A low perceived threat of COVID-19 and experiences with COVID-19 infection may also be due to the severity of the pandemic in each country. By 1 February 2022, Colombia, which has a population of 52.1 million people, had a cumulative total of 5,901,715 cases, 134,551 deaths, and a crude mortality rate of 258 deaths per 100,000 [47,48]. In contrast, Jamaica has a population of 2.9 million people and recorded 124,806 cases, 2663 deaths, and a crude mortality rate of 92 deaths per 100,000 [49]. Both countries had three waves of COVID-19 by the time of the survey, and the dominant strain was Omicron BA1 in both countries [11]. Based on the higher crude mortality rate in Colombia, it is possible that Colombia experienced more severe waves of COVID-19.

Although not well documented, the pandemic’s impact on healthcare delivery is likely to be greater in LMICs as they bear the burden of non-communicable diseases (70% of non-communicable disease deaths and 85% of premature deaths due to non-communicable diseases) and lack of resilient healthcare systems [4,50]. Our study found that most participants (>90%) in Colombia and Jamaica did not experience reduced access to prescription medications or to a primary care doctor due to service disruptions or curfews. Medication adherence was generally not affected by any COVID-19 experiences measured in our study. However, urban/rural differences were observed. For example, accessing doctors or prescription medications due to clinic or pharmacy closure was at least five times more common for participants in rural areas compared to urban areas. High levels of service disruption due to non-pharmaceutical interventions (NPIs) have been reported in other studies with important impacts on hypertension care. For example, a 2020 World Health Organization (WHO) survey of 163 member states showed that 53% of countries surveyed in the pandemic had disruption of services for hypertension treatment [51]. Other studies reported reduced access to prescription medications and medication adherence, as well as delayed diagnoses for persons with non-communicable diseases [52,53]. Shah et al. showed that uncontrolled hypertension increased from 15% to 19% during the pandemic in the US [54]. A more detailed assessment of the impact of NPI on healthcare delivery is needed to avoid interruption in care and adverse health outcomes for patients with hypertension and other chronic diseases.

Our study has important strengths, including providing information on COVID-19 experiences from a relatively large sample of hypertensive patients in two LAC countries. Although LMICs are disproportionately affected by non-communicable diseases such as hypertension and COVID-19, few studies record vaccine uptake and COVID-19 experiences in these countries. Understanding the experiences of persons with hypertension during the pandemic will provide important information to prepare for future shocks to the healthcare system, including epidemics like COVID-19 and Chikungunya. Studies documenting patient experiences in different LMIC settings are of particular value in understanding how local factors impacted these experiences. While there is documentation of differences in vaccine uptake in racial/ethnic minority populations in the US, there are limited data on these experiences in many of the source populations to which these ethnic groups belong.

However, this study has some limitations. Colombia and Jamaica used different patient recruitment strategies and recruitment of participants was slower for Jamaica. This may have resulted in some differences in country-level samples. However, we do not anticipate this would have resulted in the significantly large differences we have observed between Colombia and Jamaica in survey responses. We also relied on self-reported COVID-19 infection and vaccination, which may have resulted in some misclassification. In addition, some documented predictors of vaccination (e.g., trust in government and source of vaccine information) and important COVID-19 experiences (e.g., use of Telehealth and access to other diagnostic and screening tests) were not captured. Information was also not captured on other factors associated with a lack of fear of COVID-19 infection, such as historical and sociocultural influences. Objectively assessed availability of vaccines and access was not captured. Although we reported low levels of service disruption affecting prescribed medications, the quality of health services and subsequent health outcomes for persons with hypertension need to be assessed further. Additionally, vaccine uptake and recall of COVID-19 experiences are time-dependent and may have changed given the dynamic nature of the pandemic response. Finally, while the findings may not be generalizable to other countries outside the LAC region, they still provide important information for improving vaccine uptake and understanding healthcare disruptions in future pandemics, particularly in LMICs.

## 5. Conclusions

Differences between countries in access to rapid testing and the severity of the pandemic are possible explanations for different experiences with COVID-19 infection, fear of contracting COVID-19, and COVID-19 vaccine uptake among patients with hypertension in Colombia and Jamaica. Ensuring equitable distribution and access to rapid testing may be important for improving vaccine uptake in this subpopulation in future pandemics or public health emergencies. Greater emphasis should be placed on community engagement, which is necessary for building trust and strengthening the implementation of national responses. A comprehensive review of the COVID-19 response and experiences in LMIC can provide valuable information for public health policy and planning in future pandemics.

## Figures and Tables

**Table 1:** Characteristics of participants (N = 576) by country (Jamaica and Colombia).

Characteristic		Overall N = 576, n (%) ^a^	Jamaica N = 288, n (%) ^a^	Colombia N = 288, n (%) ^a^	*p*-Value
Age—years (mean (SD))		64.6 (11.4)	62.5 (10.2)	66.5 (12.2)	0.001
Age, (years) n (%) ^a^	<60	234 (29.1)	128 (36.0)	106 (22.9)	0.006
	60–69	226 (30.3)	106 (31.0)	120 (29.6)	
	≥70	116 (40.6)	54 (32.9)	62 (47.5)	
Sex	Male	274 (31.5)	130 (34.4)	144 (28.9)	0.2
	Female	302 (68.5)	158 (65.6)	144 (71.1)	
Marital Status	Never Married	105 (18.8)	92 (34.0)	13 (5.5)	<0.001
	Married/Common Law	323 (50.3)	130 (40.6)	193 (58.9)	
	Widowed	65 (17.0)	27 (12.4)	38 (21.1)	
	Divorced	25 (5.8)	14 (4.9)	11 (6.6)	
	Other	58 (8.0)	25 (8.1)	33 (7.9)	
Location	Urban	290 (68.1)	146 (66.0)	144 (69.9)	0.4
	Rural	286 (31.9)	142 (34.0)	144 (30.1)	
Education	Less than High school ^c^	215 (37.9)	121 (43.2)	94 (33.2)	0.01
	High school ^c^	227 (37.9)	121 (39.6)	106 (36.5)	
	More than High school ^c^	134 (24.1)	46 (17.2)	88 (30.3)	
Occupation	Employed/retired	411 (69.7)	214 (76.1)	197 (64.0)	<0.001
	Unemployed	66 (9.7)	42 (14.0)	24 (6.0)	
	Housewife	65 (15.8)	5 (1.5)	60 (28.5)	
	Disabled	34 (4.7)	27 (8.4)	7 (1.4)	
Comorbidities	Diabetes	168 (26.4)	116 (38.3)	52 (15.9)	<0.001
	High cholesterol	288 (52.7)	159 (58.9)	129 (47.1)	0.03
	Kidney Disease	18 (3.6)	4 (1.1)	14 (5.8)	0.001 ^b^
	Stroke	30 (4.9)	25 (7.7)	5 (2.5)	<0.001 ^b^
	Heart Attack/Heart Disease	36 (6.1)	8 (2.7)	28 (9.1)	0.008
	Overweight/Obesity	269 (42.9)	98 (31.9)	171 (52.7)	<0.001
Multi-morbidity ^d^	Yes (≥2 conditions)	249 (40.8)	132 (45.7)	117 (36.4)	0.077
	No	327 (59.2)	156 (54.3)	171 (63.6)	
Mental Health	Depression	31 (5.3)	9 (3.1)	22 (7.3)	0.064
	Anxiety	29 (5.0)	10 (3.7)	19 (6.1)	0.287
COVID-19 Vaccination Status	Vaccination	397 (70.1)	133 (46.7)	264 (90.7)	
	No Vaccination	178 (29.9)	154 (53.3)	24 (9.3)	<0.0001

^a^ The sample sizes and actual frequency numbers are unweighted but means (SDs) or percentages are weighted. ^b^ Fisher’s exact test; weights are rounded up to the nearest non-zero integer for the test. ^c^ High school = secondary school. ^d^ Presence of ≥2 of the following: diabetes, high cholesterol, kidney disease, stroke, heart attack/heart disease, and overweight/obesity.

**Table 2:** Impact of COVID-19 on healthcare access and utilization among urban and rural residents in Jamaica and Colombia.

Health Service Disruption	Overall N = 576	Jamaica			Colombia		
		Urban N = 146	Rural N = 142	*p*-Value ^b^	Urban N = 144	Rural N = 144	*p*-Value ^b^
Unable to see doctor/get prescription due to facility closure, n (%) ^a^	35 (4.9)	2 (1.6)	12 (13.0)	<0.001 ^c^	4 (2.1)	17 (10.4)	<0.001 ^c^
Unable to get prescription due to pharmacy closure, n (%) ^a^	23 (3.5)	0	7 (8.2)	<0.001 ^c^	5 (3.8)	11 (7.0)	0.07 ^c^
Less time to see a doctor or get medications due to curfew restrictions, n (%) ^a^	49 (7.5)	8 (5.5)	6 (3.9)	0.548	6 (3.8)	29 (19.5)	<0.001
Did not seek care due to fear of contracting coronavirus if you leave the house, n (%) ^a^	217 (39.6)	9 (6.6)	10 (7.5)	0.802	87 (61.1)	111 (79.8)	0.004
Loss of insurance or government assistance, n (%) ^a^	8 (1.2)	3 (1.2)	1 (0.8)	0.647 ^c^	2 (0.7)	2 (1.0)	0.7 ^c^
Trouble affording medications or healthcare costs, n (%) ^a^	28 (4.5)	14 (7.7)	6 (4.2)	0.216	2 (1.6)	6 (4.8)	0.06 ^c^

^a^ The sample sizes and actual frequency numbers are unweighted, but percentages are weighted. ^b^ *p*-value for rural/urban difference. ^c^ Fisher’s exact test; weights are rounded up to the nearest non-zero integer for the test.

**Table 3:** COVID-19 infection and vaccination uptake by country of residence (Jamaica and Colombia.

	Overall N = 576	Jamaica N = 288	Colombia N = 288	*p* Value
Tested positive for COVID-19, n (%) ^a^	96 (15.8)	22 (6.3)	74 (24.2)	<0.001
Family member or close friend tested positive for COVID-19, n (%) ^a^	225 (39.0)	71 (21.6)	154 (54.5)	<0.001
Loss of a family member or close friend due to COVID-19, n (%) ^a^	85 (15.1)	23 (7.8)	62 (21.5)	<0.001
Vaccinated against COVID-19, n (%)	397 (70.1)	133 (46.7)	264 (90.7)	<0.001
Among those not vaccinated, willingness to be vaccinated if available, n (%) ^a^	86 (42.4)	74 (44.4)	12 (32.2)	0.4
Vaccinated against COVID-19 or willingness to be vaccinated if available, n (%) ^a^	483 (82.9)	207 (70.6)	276 (93.7)	<0.001

^a^ The sample sizes and actual frequency numbers are unweighted, but percentages are weighted.

**Table 4:** Characteristics of participants (N = 576) in Jamaica and Colombia by COVID-19 vaccination status.

Characteristic		Vaccination N = 397	No Vaccination N = 178	*p*-Value
Age—years (mean (SD))		65.4 (11.6)	62.8 (10.6)	0.04
Age (years), n (%) ^a^	<60	153 (27.1)	81 (33.7)	0.3
	60–69	160 (30.1)	65 (30.4)	
	≥70	84 (42.8)	32 (35.8)	
Sex, n (%) ^a^	Male	188 (29.0)	86 (37.5)	0.08
	Female	209 (71.0)	92 (62.5)	
Marital Status, n (%) ^a^	Never Married	45 (11.3)	60 (36.6)	<0.001
	Married/Common Law	250 (56.8)	73 (35.2)	
	Widowed	48 (19.5)	16 (10.9)	
	Divorced	15 (5.1)	10 (7.5)	
	Other	39 (7.3)	19 (9.8)	
Location, n (%) ^a^	Urban	192 (66.8)	98 (71.2)	0.3
	Rural	205 (33.2)	80 (28.8)	
Education, n (%) ^a^	Less than High school ^c^	144 (37.3)	70 (39.2)	0.3
	High school ^c^	150 (36.3)	77 (41.9)	
	More than High school ^c^	103 (26.4)	31 (18.9)	
Occupation, n (%) ^a^	Employed/retired	285 (68.9)	126 (71.9)	<0.001
	Unemployed	40 (7.4)	26 (15.2)	
	Housewife	56 (20.7)	8 (4.1)	
	Disabled	16 (3.0)	18 (8.8)	
Comorbidities, n (%) ^a^	Diabetes	104 (23.7)	64 (32.9)	0.06
	High cholesterol	196 (52.2)	91 (53.6)	0.8
			
			
	Kidney Disease	16 (3.8)	2 (3.1)	0.3 ^b^
	Stroke	12 (3.5)	18 (8.2)	0.07
	Heart Attack/Heart Disease	28 (6.1)	8 (5.9)	0.9
	Overweight/Obesity	203 (47.4)	65 (32.3)	0.006
Multi-morbidity, n (%) ^d^	Yes (≥2 conditions)	167 (38.2)	81 (46.7)	0.138
	No	230 (61.8)	97 (53.3)	
Mental Health	Depression	20 (4.7)	11 (6.7)	0.423
	Anxiety	21 (5.0)	8 (5.2)	0.927

^a^ The sample sizes and actual frequency numbers are unweighted, but percentages are weighted. ^b^ Fisher’s exact test; weights are rounded up to the nearest non-zero integer for the test. ^c^ High school = secondary school. ^d^ Presence of diabetes, high cholesterol, kidney disease, stroke, heart attack/heart disease, and overweight/obesity.

**Table 5:** Determinants of COVID-19 vaccination uptake in patients with hypertension in Colombia and Jamaica.

Characteristic		Crude OR (95% CI)	Adjusted OR (95% CI)
Age (per 1 year increase)		1.02 (1.00–1.04)	1.01 (0.99–1.03)
Sex	Male	Ref	Ref
	Female	1.47 (0.95–2.27)	1.33 (0.75–2.35)
Education	Less than High school ^a^	Ref	Ref
	High school ^a^	0.91 (0.54–1.53)	0.79 (0.39–1.61)
	More than High school ^a^	1.47 (0.79–2.72)	0.97 (0.44–2.15)
Location	Urban	Ref	Ref
	Rural	1.23 (0.80–1.88)	1.36 (0.81–2.29)
COVID-19 infection	No COVID-19 infection or infection of family member	Ref	Ref
	COVID-19 infection or infection of family member	2.75 (1.73–4.37)	1.41 (0.82–2.42)
Complacency	No Fear of COVID-19 infection	Ref	Ref
	Fear of COVID-19 infection	9.38 (5.22–16.84)	2.71 (1.20–6.13)
Country	Jamaica	Ref	Ref
	Colombia	11.06 (5.85–20.90)	5.88 (2.38–14.56)

^a^ High school = secondary school.

## Data Availability

The original contributions presented in the study are included in the article; further inquiries can be directed to the corresponding author.

## Supplementary Materials

The following supporting information can be downloaded at: https://www.mdpi.com/article/10.3390/ijerph21101356/s1, Table S1: Characteristics of Participants (N = 287) in Jamaica by COVID-19 Vaccination Status Table S2: Characteristics of Participants (N = 288) in Colombia by COVID-19 Vaccination Status.
