# Supplementary material for: Differences in COVID-19 Vaccination and Experiences among Patients with Hypertension in Colombia and Jamaica during the COVID-19 Pandemic
Source: Int J Environ Res Public Health. 2024 Oct 15;21(10):1356. doi: 10.3390/ijerph21101356 (PMC11507612; doi:10.3390/ijerph21101356)
Supplement: Supplementary file 1 [file ijerph-21-01356-s001.zip › ijerph-3105472-supplementary.pdf]

Supplementary Materials: The following supporting information can be downloaded at: www.mdpi.com/xxx/s1, Table S1: Characteristics of Participants (N=287) in Jamaica by COVID-19 Vaccination Status; Table S2: Characteristics of Participants (N=288) in Colombia by COVID-19 Vaccination Status

Supplementary Table S1. Characteristics of Participants (N=287) in Jamaica by COVID-19 Vaccination Status

Characteristic		Vaccination N=133	No Vaccination N=154	P-value
Age – years (mean (SD))		63.0 (9.9)	60.7 (10.9)	0.163
Age (years), n (%)[a]	<60	49 (33.5)	79 (44.5)	0.204
	60-69	56 (33.6)	49 (27.5)	
	≥ 70	28 (32.9)	26 (28.0)	
Sex, n (%)[a]	Male	54 (20.0)	76 (26.6)	0.167
	Female	79 (80.0)	78 (73.4)	
Marital Status, n (%)[a]	Never Married	36 (27.8)	56 (43.3)	0.040
	Married/ Common Law	67 (48.9)	63 (32.5)	
	Widowed	13 (13.0)	13 (10.4)	
	Divorced	9 (4.9)	5 (2.2)	
	Other	8 (5.4)	17 (11.6)	
Location, n (%)[a]	Urban	59 (49.5)	87 (61.8)	0.105
	Rural	74 (50.5)	67 (38.2)	
Education, n (%)[a]	Less than High school[c]	58 (43.9)	62 (36.3)	0.474
	High school[c]	55 (41.3)	66 (42.6)	
	More than High school[c]	20 (14.8)	26 (21.1)	
Occupation, n (%)[a]	Employed/retired	105 (80.4)	109 (73.1)	0.142 [b]
	Unemployed	17 (12.9)	25 (14.7)	
	Housewife	2 (1.6)	2 (1.3)	
	Disabled	9 (5.1)	18 (10.9)	
Comorbidities, n (%)[a]	Diabetes	56 (38.9)	60 (38.5)	0.957
	High cholesterol	78 (66.4)	80 (55.4)	0.132
	Kidney Disease	3 (1.7)	1 (0.3)	0.201 [b]
	Stroke	8 (5.2)	17 (7.3)	0.450
	Heart Attack/Heart Disease	5 (4.8)	3 (1.4)	0.161 [b]
	Overweight/Obesity	44 (33.3)	53 (36.8)	0.631
Multi-morbidity, n (%)[a,d]	Yes (≥2 conditions)	59 (44.1)	72 (50.6)	0.400
	No	74 (55.9)	82 (49.4)	
Mental Health	Depression	3 (2.3)	6 (4.2)	0.263 [b]
	Anxiety	5 (3.8)	5 (3.9)	0.976

[a] The sample sizes and actual frequency numbers are unweighted, but percentages are weighted.
[b] Fisher's exact test method, weights are rounded up to the nearest non-zero integer for test.
[c] High school = secondary school
[d] Presence of ≥2 of the following: diabetes, high cholesterol, kidney disease, stroke, heart attack/ heart disease, and overweight/obesity

Supplementary Table S2. Characteristics of Participants (N=288) in Colombia by COVID-19 Vaccination Status

Characteristic		Vaccination N=264	No Vaccination N=24	P-value
Age – years (mean (SD))		67.4 (11.9)	68.5 (5.6)	0.466
Age (years), n (%)[a]	<60	104 (21.1)	2 (1.9)	< 0.001 [b]
	60-69	104 (29.3)	16 (35.0)	
	≥ 70	56 (49.6)	6 (63.2)	
Sex, n (%)[a]	Male	134 (38.9)	10 (46.1)	0.637
	Female	130 (61.1)	14 (53.9)	
Marital Status, n (%)[a]	Never Married	9 (5.0)	4 (22.4)	< 0.001 [b]
	Married/ Common Law	183 (62.3)	10 (41.8)	
	Widowed	35 (21.7)	3 (13.0)	
	Divorced	6 (4.5)	5 (20.5)	
	Other	31 (6.5)	2 (2.3)	
Location, n (%)[a]	Urban	133 (80.2)	11 (83.9)	0.609
	Rural	131 (19.8)	13 (16.1)	
Education, n (%)[a]	Less than High school[c]	86 (29.6)	8 (27.0)	0.306
	High school[c]	95 (33.9)	11 (54.8)	
	More than High school[c]	83 (36.5)	5 (18.2)	
Occupation, n (%)[a]	Employed/retired	180 (68.1)	17 (73.4)	0.581 [b]
	Unemployed	23 (6.3)	1 (13.7)	
	Housewife	54 (23.4)	6 (12.8)	
	Disabled	7 (2.2)	0	
Comorbidities, n (%)[a]	Diabetes	48 (15.8)	4 (7.1)	0.404 [b]
	High cholesterol	118 (47.4)	11 (56.5)	0.554
	Kidney Disease	13 (4.6)	1 (13.7)	0.092 [b]
	Stroke	4 (2.3)	1 (0.8)	> 0.999 [b]
	Heart Attack/Heart Disease	23 (9.9)	5 (30.3)	0.207
	Overweight/Obesity	159 (52.6)	12 (29.5)	0.130
Multi-morbidity, n (%))[a,d]	Yes (≥2 conditions)	108 (35.5)	9 (39.2)	0.803
	No	156 (64.5)	15 (60.8)	
Mental Health	Depression	17 (5.5)	5 (20.8)	0.192
	Anxiety	16 (4.8)	3 (15.1)	0.021 [b]

[a] The sample sizes and actual frequency numbers are unweighted, but percentages are weighted.
[b] Fisher's exact test method, weights are rounded up to the nearest non-zero integer for test.
[c] High school = secondary school
[d] Presence of ≥2 of the following: diabetes, high cholesterol, kidney disease, stroke, heart attack/ heart disease, and overweight/obesity
